# The New Role of CD163 in the Differentiation of Bone Marrow Stromal Cells into Vascular Endothelial-Like Cells

**DOI:** 10.1155/2016/2539781

**Published:** 2016-01-06

**Authors:** Wei Lu, Le Su, Zhezheng Yu, Shangli Zhang, Junying Miao

**Affiliations:** ^1^Shandong Provincial Key Laboratory of Animal Cells and Developmental Biology, School of Life Science, Shandong University, Jinan 250100, China; ^2^The Key Laboratory of Cardiovascular Remodeling and Function Research, Chinese Ministry of Education and Chinese Ministry of Health, Shandong University Qilu Hospital, Jinan 250012, China

## Abstract

Bone marrow stromal cells (BMSCs) can differentiate into vascular endothelial cells (VECs). It is regarded as an important solution to cure many diseases, such as ischemic diseases and diabetes. However, the mechanisms underlying BMSC differentiation into VECs are not well understood. Recent reports showed that CD163 expression was associated with angiogenesis. In this study, overexpression of CD163 in BMSCs elevated the protein level of the endothelial-associated markers CD31, Flk-1, eNOS, and VE-cadherin, significantly increased the proportion of Alexa Fluor 488-acetylated-LDL-positive VECs, and promoted angiogenesis on Matrigel. Furthermore, we demonstrated that CD163 acted downstream homeobox containing 1 (Hmbox1) and upstream fibroblast growth factor 2 (FGF-2). These data suggested that CD163 was involved in Hmbox1/CD163/FGF-2 signal pathway in BMSC differentiation into vascular endothelial-like cells. We found a new signal pathway and a novel target for further investigating the gene control of BMSC differentiation into a VEC lineage.

## 1. Introduction

Bone marrow stromal cells (BMSCs), due to their low immunogenicity and multilineage differentiation potential, promise as a main actor of cell-based therapeutic strategies [1]. They are able to differentiate into various kinds of cells, including vascular endothelial cells (VECs), osteocytes, chondrocytes, adipocytes, and neural cells [2–4]. VECs have been shown to play a central role in vasculogenesis during development and angiogenesis [5]. After being implanted into vein grafting in rats, these BMSCs differentiate into VECs and inhibit advanced vascular lesion formation [6]. Therefore, BMSCs' transplantation is regarded as an important strategy to ameliorate much damage or cure diseases, including spinal cord injury, disc degeneration, cerebral ischemic disease, and diabetes [7]. However, the mechanism of BMSC differentiation into VECs is not well-known.

BMSCs can be induced to differentiate into VECs under certain conditions* in vitro* [2, 8–10]. (1) The classical proangiogenic inducer VEGF, FGF-2, and PDGF could turn on this differentiation through MAKP signaling pathway [8]. (2) Simvastatin enhances this process via notch signaling pathway [9]. (3) Our previous study showed that chemical small molecule, 6-amino-2,3-dihydro-3-hydroxymethyl-1,4-benzoxazine (ABO), could promote BMSC/mouse embryonic stem cell (mESC) differentiation into VECs by elevating homeobox containing 1 (Hmbox1) expression [2, 10].

CD163 was first identified in 1987 and received its CD number in 1996 [11, 12]. It was widely thought that expression of CD163 is restricted to cells of the monocyte/macrophage lineage [13, 14]. However, recent analyses have revealed that the tumor cell itself in breast, rectal, and bladder cancer expressed CD163 [15]. Moreover, CD163 is also highly expressed in endothelial progenitor cells (EPCs) [16]. Since EPCs have been shown to be involved in neovascularization and angiogenesis, it is suggested that CD163 may be associated with endothelial differentiation.

CD163 belongs to the cysteine-rich scavenger receptor superfamily type B [11]. Most studies focus on inflammation. In monocyte/macrophage lineage, expression of CD163 is tightly regulated, with a general tendency of anti-inflammatory signals to induce CD163 synthesis, while proinflammatory signals rather seem to downregulate CD163 expression [17]. The first-identified and most-studied function of CD163 is related to its capacity to bind and internalize haemoglobin-haptoglobin (HbHp) complexes [17, 18]. Later on, its functional repertoire was expanded, with the identification of CD163 as a receptor for tumour necrosis factor-like weak inducer of apoptosis (TWEAK), which is a TNF superfamily member and mediates angiogenesis [19–22]. Furthermore, recent report showed that CD163 expression is associated with angiogenesis [23]. Therefore, although CD163 has been most extensively studied in immunoregulatory context, there are many clues suggesting the relationship between CD163 and endothelial differentiation.

In our previous study, we found that the level of Hmbox1 was upregulated in the differentiation of BMSC/mESC into VECs induced by ABO. Moreover, ABO failed to induce the formation of VECs without Hmbox1 [2, 10]. Hence, Hmbox1 was a key factor in BMSC/mESC differentiation into VECs. Furthermore, Hmbox1 acted upstream the classical FGF-2 proangiogenic pathway in ABO-induced mESCs differentiation into VECs [10]. However, the relationship between CD163 and Hmbox1/FGF-2 signaling in BMSC differentiation into VECs was unknown. In this study, we demonstrated that CD163 was positively regulated by Hmbox1, and it could promote BMSC differentiation into vascular endothelial-like cells through enhancing FGF-2 signaling.

## 2. Materials and Methods

### 2.1. Cell Culture

Rat BMSCs were isolated from the femurs and tibias of male Wistar rats (90–100 g) as described earlier with modification [24]. Rats were killed by intravenous injection of ketamine/xylazine, and all work was performed according to the Institutional Animal Care and Use Committee guidelines. This experimental protocol was reviewed and approved by the Medical Ethics Committee of Shandong University School of Medicine. Briefly, the cells were seeded in Dulbecco's modified Eagle's medium low glucose (Gibco, USA) supplemented with 15% fetal bovine serum and fibroblast growth factor 2 (FGF-2) (Beifuji, China), 10 ng/mL, at 37°C in humidified air with 5% CO_2_. Rat BMSCs were phenotypically characterized as described [25]. Cells were transfected with pCMV6 empty vector/pCMV6-CD163 in DMEM with FGF-2 (10 ng/mL), rVEGF (10 ng/mL) (Sigma, St. Louis, MO), and 2% serum and incubated at 37°C for 24 h. Then, the medium is replaced by basal DMEM with FGF-2 (10 ng/mL) and rVEGF (10 ng/mL) incubated at 37°C for another 24 h.

### 2.2. Plasmids and Overexpression

BMSCs were cultured to 80% confluent prior to transfection in basal DMEM and transient transfection was performed using Lipofectamine 2000 (Invitrogen, 11668-019) transfection reagent according to the manufacturer's instructions. Cells were transfected with pCMV6-CD163 (Origene, RN206586), pCMV6-Hmbox1 (Origene, SC319800), or pCMV6 empty vector.

### 2.3. Alexa 488-Ac-LDL Uptake Assay

Cells were seeded on 24-well culture plates and incubated in 10 *μ*g/mL Alexa Fluor 488-acetylated low-density lipoprotein (Alexa 488-Ac-LDL) (Invitrogen, USA) at 37°C for 4 to 6 h. The medium was removed and cells were washed once with the culture medium. The cells that could uptake Alexa 488-Ac-LDL showed green fluorescence with LSCM excitation at 488 nm. The ability to uptake Alexa 488-Ac-LDL was used to estimate the differentiation rate of BMSCs: the number of positively stained cells divided by the total number of cells in random visual fields. At least 200 cells for each sample were counted.

### 2.4. *In Vitro* Capillary-Like Tube Formation Assay

The formation of vascular-like structures in BMSCs was assessed by the use of Matrigel (YEASEN Biosciences, China) as previously described [26]. Briefly, cells were seeded on 24-well plates coated with Matrigel at 4-5 *∗* 10^4^ cells per well within DMEM with 15% serum and incubated at 37°C for 60–90 min. Then, cell culture medium was replaced by the basal DMEM. Finally, after 4–7 hours of initial cell seeding, tube formation was observed under an inverted-phase contrast microscope (Nikon, Japan).

### 2.5. RNA Interference

To knock down the expression of CD163 or Hmbox1, RNA interference (RNAi) was performed as described [27]. BMSCs were cultured to 50–60% confluence prior to transfection in basal DMEM, and transient transfection was performed using HiPerFect (Qiagen, 301704) transfection reagent according to the manufacturer's instructions. Cells were transfected with siRNA for CD163 (Origene, SR506300) or Hmbox1 (designed and synthesized by Invitrogen, USA). The synthesized siRNA to Hmbox1 was (RNA)-UUU CAG AGA CGU AAC UCG UUC CAG G (sense) and (RNA)-CCU GGA ACG AGU UAC GUC UCU GAA A (antisense). Scramble siRNA was used as a control (Santa Cruz Biotechnology, USA).

### 2.6. Immunofluorescence Assay

Immunofluorescence assay was performed as described previously [28]. After adding the primary antibodies, rabbit anti-eNOS IgG (Santa Cruz Biotechnology, sc-9989 1 : 100) or mouse anti-VE-cadherin IgG (Santa Cruz Biotechnology, sc-654 1 : 100), and appropriate secondary antibodies (fluorescein isothiocyanate- (FITC) - donkey anti-rabbit/mouse IgG; all Biotechnology, Santa Cruz, CA), the samples were evaluated by laser scanning confocal microscopy (LSCM) (Leica, Germany). eNOS displayed green fluorescence after excitation at 488 nm and VE-cadherin displayed red fluorescence after excitation at 546 nm. We randomly selected the region of interest and then zoomed in the same frames. The relative fluorescent intensity (analyzed by ImageJ software) per cell was the total value of the sample in the zoom scan divided by the total number of cells (at least 200 cells) in the same scan.

### 2.7. Western Blot Analysis

Western blot was performed as described previously [29]. Briefly, the cells were lysed in protein lysis buffer containing 25 mM Tris-HCl (pH 6.8), 2% SDS, 6% glycerol, 1% 2-mercaptoethanol, 2 mM PMSF, 0.2‰ bromophenol blue, and a protease inhibitor cocktail (Sigma, St. Louis, MO) for 10 min at RT (room temperature) and boiled for another 10 min. The protein concentration was determined by Coomassie brilliant blue protein assay. The cellular proteins (20~40 *μ*g) were applied to 15% or 9% SDS polyacrylamide gel and electroblotted onto polyvinylidene difluoride (PVDF) membrane. The membrane was blocked with 5% (w/v) nonfat dry milk in phosphate buffered saline (PBS), Tween 20 (PBST; 0.05%) for 1 h and incubated with anti-CD163 (Santa Cruz Biotechnology, sc-58965 1 : 100), anti-Hmbox1 (Santa Cruz Biotechnology, sc-87768 1 : 1000), anti-FGF-2 (Santa Cruz Biotechnology, sc-271847 1 : 1000), or anti-*β*-actin antibody (Santa Cruz Biotechnology, sc-47778 1 : 1000) at 4°C overnight. After washing in PBST and PBS, the PVDF membrane was incubated with appropriate horseradish peroxidase-conjugated secondary antibodies (Santa Cruz Biotechnology 1 : 10000) for 1 h at RT. The membrane was incubated with Immobilon Western Chemiluminescent HRP Substrate (Millipore, WBKLS0500) for 5 min at RT and exposed to X-ray film (Kodak). The relative quantity of proteins was analyzed by the use of ImageJ software and normalized to loading controls.

### 2.8. Statistical Analyses

Data are expressed as mean ± SEM. Images were processed by use of Adobe Photoshop CS4 (Adobe, San Jose, USA). SPSS 20.0.0 (SPSS Inc., Chicago, IL) was used for statistical analysis. Comparisons among groups involved one-way ANOVA followed by Scheffé  *F*-test post hoc analysis. A *p* < 0.05 was considered statistically significant.

## 3. Results

### 3.1. CD163 Promoted BMSC Differentiation into Vascular Endothelial-Like Cells

**Figure 1:**
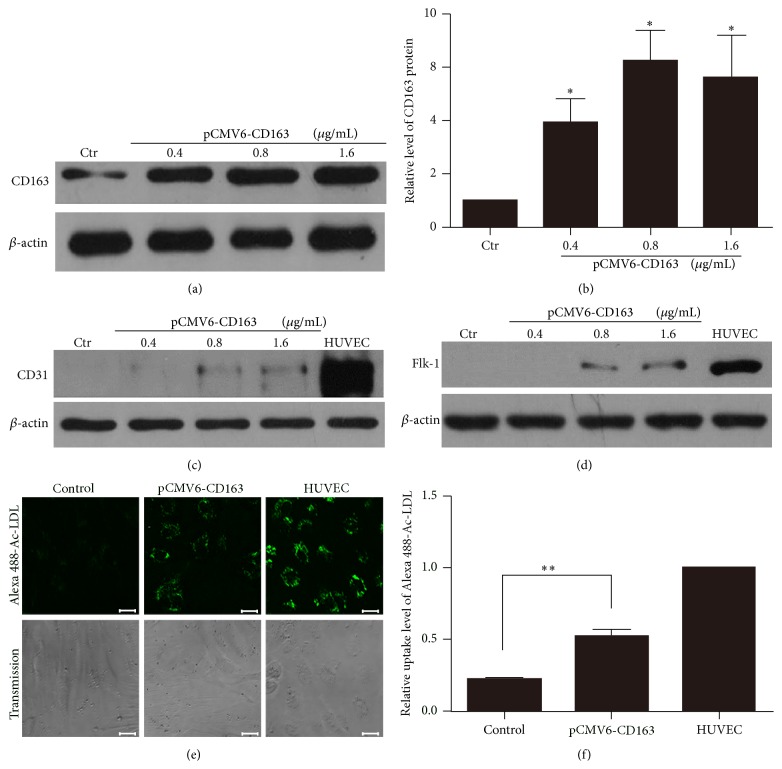
Overexpression of CD163 enhanced the expression of endothelial-associated markers and uptake of Alexa 488-Ac-LDL in BMSCs. For CD163 overexpression, BMSCs were transfected with empty vector pCMV6 (as control) at 1.6 *μ*g/mL or pCMV6-CD163 at 0.4, 0.8, or 1.6 *μ*g/mL for 4 h and cultured for 48 h. Ctr: control. (a) Western blot analysis of CD163 overexpression efficiency. (b) Relative levels of CD163 protein were calculated based on densitometry analysis. ((c)-(d)) Western blot analysis of the protein level of the endothelial-associated markers CD31 and Flk-1 after being transfected with pCMV6 or pCMV6-CD163 for 48 h. (e) Uptake of Alexa 488-Ac-LDL by cells after being transfected with pCMV6 or pCMV6-CD163 at 0.8 *μ*g/mL for 48 h. Scale bar: 10 *μ*m. Images are representative of at least 3 independent experiments. (f) Relative uptake level of Alexa 488-Ac-LDL was calculated by LCS lite. The uptake level of HUVEC was set as 1. HUVEC: human umbilical vascular endothelial cells as a positive control. Data are mean ± SEM. ^*∗*^ *p* < 0.05, ^*∗∗*^ *p* < 0.01 versus control (Ctr), *n* ≥ 3.

Since many studies suggested the relationship between CD163 and endothelial differentiation, we intend to investigate the role of CD163 in the differentiation of BMSCs to VECs. The full-length cDNA sequence of CD163 was cloned into the pCMV6 expression vector, pCMV6-CD163. BMSCs were transfected with pCMV6 empty vector at 1.6 *μ*g/mL or pCMV6-CD163 at 0.4, 0.8, and 1.6 *μ*g/mL for 48 h with rVEGF and FGF-2. The efficiency of overexpression CD163 was confirmed by western blotting. Compared with pCMV6 empty vector, pCMV6-CD163 increased the protein level of CD163 in BMSCs in a dose-dependent manner (Figures 1(a) and 1(b)). To confirm the role of CD163 in the endothelial differentiation of BMSCs, we detected the protein level of the endothelial-associated markers CD31 and Flk-1 by western blotting after being transfected with pCMV6-CD163 or pCMV6 empty vector for 48 h in BMSCs. Human umbilical vein endothelial cells (HUVECs) were set as a VECs positive control. The results showed that protein levels of the endothelial-associated markers CD31 and Flk-1 were undetectable in BMSCs transfected with pCMV6 empty vector, but they were observed in BMSCs transfected with pCMV6-CD163 at 0.8, 1.6 *μ*g/mL (Figures 1(c) and 1(d)). It is suggested that CD163 promoted the expression of endothelial-associated markers CD31 and Flk-1 in BMSCs.

VECs are functionally defined by their uptake capacity to acetylated low-density lipoprotein from plasma. For further investigating the differentiation rate and defining the function of CD163 overexpression BMSC-derived VECs, we detected a number of Alexa Fluor 488-acetylated low-density lipoprotein-positive VECs in CD163 overexpression BMSCs. As shown in Figures 1(e) and 1(f), overexpression of CD163 significantly increased the number of Alexa Fluor 488-acetylated-LDL-positive cells. HUVECs were set as a VEC positive control. The differentiation proportion of BMSCs was 52.6% after transfection with pCMV6-CD163, whereas the proportion of the control group was only 22%. This result showed that CD163 overexpression in BMSCs effectively enhanced the uptake of Alexa Fluor 488-acetylated low-density lipoprotein (Alexa 488-Ac-LDL).

**Figure 2:**
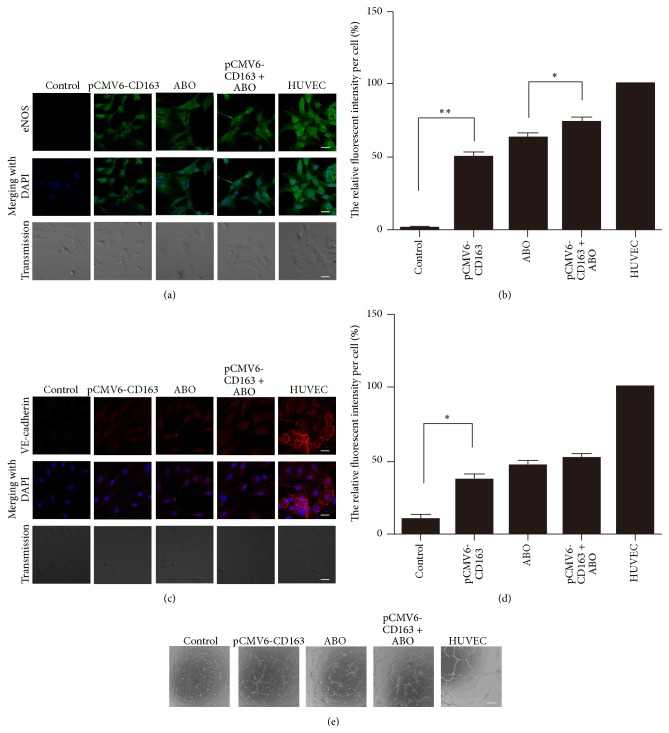
CD163 overexpression promoted BMSC differentiation into vascular endothelial-like cells with or without ABO treatment. In control group, BMSCs were transfected with empty vector pCMV6 and treated with 0.05% DMSO for 48 h; in pCMV6-CD163 group, BMSCs were transfected with pCMV6-CD163 for 48 h; in ABO group, BMSCs were treated with 50 *μ*M ABO for 48 h (DMSO as control); in pCMV6-CD163 + ABO group, BMSCs were transfected with pCMV6-CD163 and treated with 50 *μ*M ABO for 48 h; and in HUVEC group, human umbilical vascular endothelial cells are considered as a positive control. (a) Immunostaining of the endothelial-function associated marker endothelial nitric oxide synthase (eNOS) in BMSCs treated as described above. Scale bar: 10 *μ*m. (b) The relative fluorescent intensity was calculated and the fluorescent intensity of HUVECs was set as 100%. (c) Immunostaining of the endothelial-specific marker VE-cadherin in BMSCs treated as described above. Scale bar: 10 *μ*m. (d) The relative fluorescent intensity was calculated and the fluorescent intensity of HUVECs was set as 100%. (e) The vascular structure formed on Matrigel by BMSCs treated as described above. Scale bar: 100 *μ*m. Images are representatives of at least 3 independent experiments. Data are mean ± SEM. ^*∗*^ *p* < 0.05, ^*∗∗*^ *p* < 0.01, *n* ≥ 3.

Furthermore, we also examined the expression of the endothelial-function associated marker endothelial nitric oxide synthase (eNOS) and the endothelial-specific marker VE-cadherin by immunofluorescence. eNOS is tightly associated with endothelial function and vascular homeostasis. Its expression was dramatically induced by CD163 overexpression in BMSCs (Figures 2(a) and 2(b)). Compared with the control group, the expression of the endothelial-specific marker VE-cadherin was induced after BMSC transfected with pCMV6-CD163 as well (Figures 2(c) and 2(d)). The data suggested that the overexpression of CD163 promoted the expression of endothelial-function associated marker eNOS and endothelial-specific marker VE-cadherin in BMSCs.

Angiogenesis is the process where new blood vessels sprout out of preexisting vessels and this process involves the recruitment and proliferation of VEC [30, 31].* In vitro* angiogenesis assay has shown that mature VECs form a capillary-like network when plated on Matrigel [32]. We used a Matrigel based angiogenesis assay to define the functionality of the CD163 overexpression BMSCs derived vascular endothelial-like cells and HUVECs were set as a VEC positive control. Unlike the control group, capillary-like network was observed in BMSCs transfected with pCMV6-CD163 and in HUVECs (Figure 2(e)).

Collectively, our data suggested that, compared with the control group, BMSCs with CD163 overexpression enhanced the differentiation of BMSCs into vascular endothelial-like cells.

### 3.2. CD163 Played an Important Role in ABO-Induced Differentiation of BMSCs to VECs

In our previous study, we found that ABO could induce BMSC differentiation into VECs by elevating Hmbox1 expression [2]. We wondered whether CD163 participated in ABO-induced differentiation of BMSCs to VECs. And we found that, compared with ABO group, the angiogenesis capacity and the expression of eNOS and VE-cadherin were further enhanced by CD163 overexpression and ABO treatment (Figure 2). It suggested that CD163 might be involved in ABO-induced endothelial differentiation of BMSCs.

**Figure 3:**
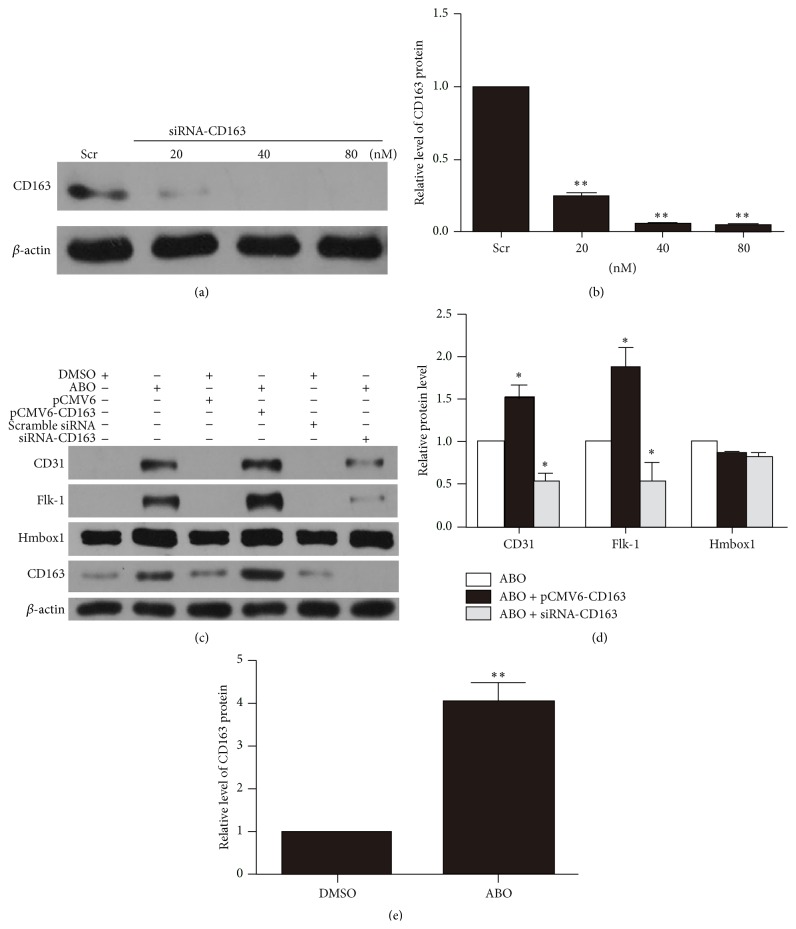
The protein level of CD163 influenced ABO-induced BMSC differentiation. For CD163 interference, cells were subjected with 80 nmol/L scrambled siRNA or 20, 40, and 80 nmol/L CD163 siRNA for 6 h and cultured for 48 h. Scr: scrambled RNA as negative control. (a) Western blot analysis of CD163 RNA interference efficiency. (b) Relative levels of CD163 protein were calculated based on densitometry analysis. (c) Western blot analysis of the protein level of CD31, Flk-1, Hmbox1, and CD163 after overexpression or knockdown of CD163 with or without 50 *μ*M ABO treatment (DMSO as control). ((d)-(e)) Relative protein levels of CD31, Flk-1, Hmbox1, and CD163 were calculated based on densitometry analysis. Data are mean ± SEM. ^*∗*^ *p* < 0.05 versus ABO group, ^*∗∗*^ *p* < 0.01 versus control (Ctr), *n* ≥ 3.

To verify whether CD163 participates in ABO-induced endothelial differentiation of BMSCs, pCMV6-CD163 or siRNA-CD163 was used to overexpress or knock down CD163 with ABO treatment, respectively. For knockdown of CD163, we detected the siRNA-CD163 efficiency in BMSCs by western blotting (Figures 3(a) and 3(b)). As shown in Figures 3(c) and 3(d), on the basis of enhanced protein level of CD31 and Flk-1 after 50 *μ*M ABO treatment for 48 h, pCMV6-CD163 further increased their protein level; it was more important that siRNA-CD163 significantly decreased their protein level. The data suggested that CD163 had an important role in ABO-induced endothelial differentiation of BMSCs.

### 3.3. CD163 Was Positively Regulated by Hmbox1 in BMSCs

**Figure 4:**
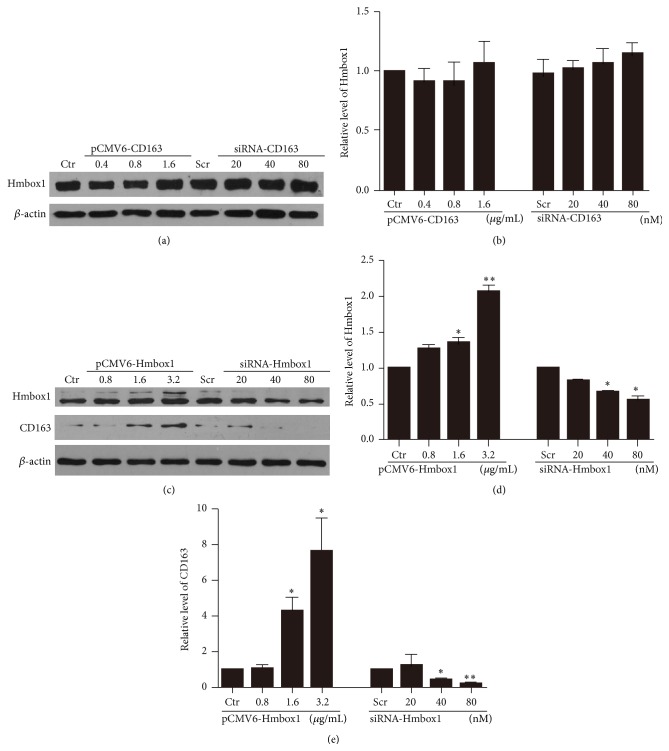
CD163 acted downstream Hmbox1 in differentiation of BMSCs towards a VEC lineage. (a) Western blot analysis of the protein level of Hmbox1 after overexpression or knockdown of CD163. (b) Relative protein levels of Hmbox1 were calculated based on densitometry analysis. (c) Western blot analysis of the protein level of Hmbox1 and CD163 after overexpression or knockdown of Hmbox1. For Hmbox1 overexpression, BMSCs were transfected with pCMV6 empty vector (as control) at 3.2 *μ*g/mL or pCMV6-Hmbox1 at 0.8, 1.6, or 3.2 *μ*g/mL for 4 h and cultured for 48 h. For Hmbox1 interference, cells were subjected with 80 nmol/L scrambled siRNA or 20, 40, and 80 nmol/L Hmbox1 siRNA for 6 h and cultured for 48 h. Scr: scrambled RNA as negative control. ((d)-(e)) Relative protein levels of Hmbox1 and CD163 were calculated based on densitometry analysis. Data are mean ± SEM. ^*∗*^ *p* < 0.05, ^*∗∗*^ *p* < 0.01 versus control (Ctr), *n* ≥ 3.

To investigate the relationship between CD163 and Hmbox1 in endothelial differentiation of BMSCs, we detected the protein level of CD163 and Hmbox1 with 50 *μ*M ABO treatment for 48 h and transfected pCMV6-CD163 or siRNA-CD163 to BMSCs at the same time. ABO treatment significantly improved the protein level of CD163 (Figures 3(c) and 3(e)), whereas changed protein level of CD163 cannot influence the protein level of Hmbox1 with ABO treatment (Figures 3(c) and 3(d)). And even without ABO treatment, transfected pCMV6-CD163 or siRNA-CD163 also cannot alter the protein level of Hmbox1 (Figures 4(a) and 4(b)). According to our previous work, we deduced that Hmbox1 was located upstream CD163 in this differentiation regulation pathway. We examined the protein level of CD163 after BMSCs were transfected with pCMV6-Hmbox1 or siRNA-Hmbox1 (Figures 4(c), 4(d), and 4(e)). Overexpression of Hmbox1 upregulated the protein level of CD163, whereas knockdown of Hmbox1 downregulated CD163 in BMSCs. These results confirmed that Hmbox1 positively regulated CD163 in BMSCs.

### 3.4. CD163 Acted Upstream FGF-2 in the Differentiation of BMSCs into a VEC Lineage

**Figure 5:**
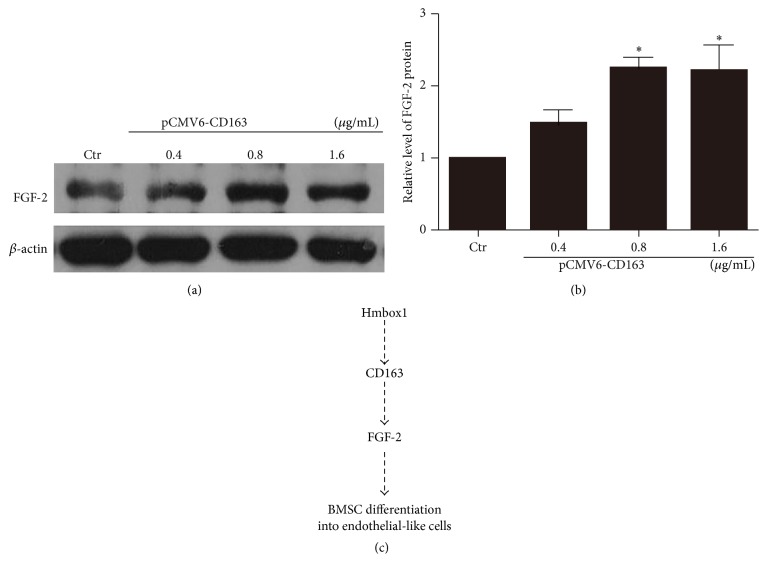
CD163 acted upstream FGF-2 in BMSC differentiation into a vascular endothelial lineage. (a) Western blot analysis of the protein level of FGF-2 after overexpression of CD163. (b) Relative protein levels of FGF-2 were calculated based on densitometry analysis. Data are mean ± SEM. ^*∗*^ *p* < 0.05, ^*∗∗*^ *p* < 0.01 versus control (Ctr), *n* ≥ 3. (c) A conceptual schematic of CD163 involved in the differentiation of BMSCs into a vascular endothelial lineage. Hmbox1 increases the expression of CD163, which promotes BMSC differentiation into vascular endothelial-like cells by enhancing FGF-2.

Our previous study showed that ABO could induce mESC differentiation into VECs by enhancing Hmbox1 expression and further upregulating the protein level of the important proangiogenic factor, FGF-2 [10]. To further understand the mechanism of CD163 in the differentiation of BMSCs to a VEC lineage, we detected the protein level of FGF-2 in BMSCs after being transfected with pCMV6-CD163 or pCMV6 empty vector for 48 h. As shown in Figures 5(a) and 5(b), compared with the control group, the protein level of FGF-2 was significantly increased in BMSCs after CD163 overexpression. These data suggested that CD163 promoted the differentiation of BMSCs into a VEC lineage through enhancing FGF-2 signaling.

## 4. Discussion

In this study, we first studied the role of CD163 in positive regulation of the differentiation of BMSC into vascular endothelial-like cells. We demonstrated as well that CD163 took part in and further promoted the endothelial differentiation process induced by ABO in BMSCs. Moreover, overexpression of Hmbox1 upregulated the protein level of CD163; meanwhile overexpression of CD163 upregulated the protein level of FGF-2. These data suggested that CD163 was involved in Hmbox1/CD163/FGF-2 signal pathway in the differentiation of BMSCs into vascular endothelial-like cells (Figure 5(c)). Collectively, the novel finding of this study was the role of CD163 in BMSC differentiation towards a VEC lineage. Meanwhile, we demonstrated that CD163 regulated the differentiation of BMSCs into vascular endothelial-like cells via Hmbox1/CD163/FGF-2 signaling (Figure 5(c)).

The hemoglobin (Hb) scavenger receptor, CD163, has a key role in the control of inflammatory processes by the induction of anti-inflammatory pathways [33]. A recent report showed that CD163 expression was associated with angiogenesis [23]; however, little is known about its role in angiogenesis. It is for the first time proposed that CD163 plays an important role in the differentiation of BMSCs into vascular endothelial-like cells. The findings of this work provided a novel target for control of BMSC differentiation into a vascular endothelial lineage and for further investigation of CD163 functions.

It is very complex that the expression of CD163 is regulated by a variety of factors. On its promoter sequence, there are one major transcription start site, six alternative initiation sites, and possible binding sites for transcription factors like glucocorticoid receptor, PU.1, C/EBP, Ets-2, and AP-1, which have been shown to play an important role in myeloid-specific gene expression [34]. There is no TATA box in its proximal sequence, which also is a common feature of many myeloid-specific promoter sequences [17, 34]. Hence, CD163 not only plays pivotal roles in monocytes and macrophages, but also may play important roles in BMSCs.

Hmbox1 was a key factor in BMSC/mESC differentiation into VECs and upregulated Ets-1 in BMSC differentiation into VECs [2, 10]. Here, we found that Hmbox1 positively regulated CD163, while CD163 might be regulated by Ets-2 [34]. Meanwhile, plenty of reports showed that Ets-1 and Ets-2 are functionally redundant in lots of cases and required for endothelial cell survival [35–38]. It is a possible pathway that CD163 is positively regulated by Hmbox1/Ets-1 signaling, and, in our further investigation, we will verify this pathway of Hmbox1/Ets-1/CD163 signaling.

In summary, our data suggested that CD163, which was positively regulated by Hmbox1 and acted upstream FGF-2, was a key regulator in this differentiation process. These findings provided a novel target for further investigating the gene control of BMSC differentiation into vascular endothelial-like cells.
